# Enhanced Antiviral Activity of Human Surfactant Protein D by Site-Specific Engineering of the Carbohydrate Recognition Domain

**DOI:** 10.3389/fimmu.2019.02476

**Published:** 2019-10-22

**Authors:** Martin van Eijk, Marine L. B. Hillaire, Guus F. Rimmelzwaan, Michael J. Rynkiewicz, Mitchell R. White, Kevan L. Hartshorn, Martin Hessing, Peter A. Koolmees, Monique H. Tersteeg, Maarten H. van Es, Tjarko Meijerhof, Anke Huckriede, Henk P. Haagsman

**Affiliations:** ^1^Division of Molecular Host Defence, Department of Infectious Diseases and Immunology, Faculty of Veterinary Medicine, Utrecht University, Utrecht, Netherlands; ^2^Department of Viroscience, Erasmus MC, Rotterdam, Netherlands; ^3^Research Center for Emerging Infections and Zoonoses, University of Veterinary Medicine, Hanover, Germany; ^4^Department of Physiology and Biophysics, Boston University School of Medicine, Boston, MA, United States; ^5^Department of Medicine, Boston University School of Medicine, Boston, MA, United States; ^6^U-Protein Express B.V., Life Science Incubator, Utrecht, Netherlands; ^7^Division of Veterinary Public Health, Faculty of Veterinary Medicine, Institute for Risk Assessment Sciences, Utrecht University, Utrecht, Netherlands; ^8^NOMI, Optomechatronics, TNO, Delft, Netherlands; ^9^Department of Medical Microbiology, University of Groningen, University Medical Center Groningen, Groningen, Netherlands

**Keywords:** antiviral agent, influenza A virus, innate immunity, surfactant protein D, collectin, pandemics, recombinant expression, lung infection

## Abstract

Innate immunity is critical in the early containment of influenza A virus (IAV) infection and surfactant protein D (SP-D) plays a crucial role in innate defense against IAV in the lungs. Multivalent lectin-mediated interactions of SP-D with IAVs result in viral aggregation, reduced epithelial infection, and enhanced IAV clearance by phagocytic cells. Previous studies showed that porcine SP-D (pSP-D) exhibits distinct antiviral activity against IAV as compared to human SP-D (hSP-D), mainly due to key residues in the lectin domain of pSP-D that contribute to its profound neutralizing activity. These observations provided the basis for the design of a full-length recombinant mutant form of hSP-D, designated as “improved SP-D” (iSP-D). Inspired by pSP-D, the lectin domain of iSP-D has 5 amino acids replaced (Asp324Asn, Asp330Asn, Val251Glu, Lys287Gln, Glu289Lys) and 3 amino acids inserted (326Gly-Ser-Ser). Characterization of iSP-D revealed no major differences in protein assembly and saccharide binding selectivity as compared to hSP-D. However, hemagglutination inhibition measurements showed that iSP-D expressed strongly enhanced activity compared to hSP-D against 31 different IAV strains tested, including (pandemic) IAVs that were resistant for neutralization by hSP-D. Furthermore, iSP-D showed increased viral aggregation and enhanced protection of MDCK cells against infection by IAV. Importantly, prophylactic or therapeutic application of iSP-D decreased weight loss and reduced viral lung titers in a murine model of IAV infection using a clinical isolate of H1N1pdm09 virus. These studies demonstrate the potential of iSP-D as a novel human-based antiviral inhalation drug that may provide immediate protection against or recovery from respiratory (pandemic) IAV infections in humans.

## Introduction

Influenza is an infectious respiratory disease, caused by influenza A viruses (IAVs), that affects a wide range of natural hosts including humans, pigs and birds. Worldwide, annual epidemics result in 3 to 5 million cases of severe illness and about 300.000 to 650.000 deaths (1). Moreover, novel IAVs have potential to cause influenza pandemics like the most recent outbreak in 2009 that was estimated to have caused between 100.000 and 400.000 deaths globally in the first year alone (2). Pigs, in particular, are susceptible to co-infection by both avian and human IAVs and as a consequence, novel reassorted IAVs can be introduced to the human population as illustrated by the swine-origin pandemic flu of 2009 (3). During the early stages of IAV respiratory infection, the innate immune response plays a key role in limiting IAV replication and thereby spread of infection, modulating the inflammatory and adaptive responses. Distinct responses cause the differences in pathogenicity that result from infection by pandemic vs. seasonal IAVs (4). Given the important role of pigs in being susceptible for co-infection by a broad range of IAVs and since persistence of infection readily occurs in this animal species (5), studies were initiated to investigate porcine pulmonary innate responses against IAV in more detail.

Pulmonary innate defense involves the secretion of soluble proteins that are capable of neutralizing IAV infectivity, several of which are members of the “collectin” family (6). These collagenous lectins include surfactant protein D (SP-D), a large cruciform-shaped glycoprotein that exhibits strong antiviral properties, as demonstrated by many *in vitro* and *in vivo* studies [reviewed in (7)]. The dodecameric X-shaped configuration of SP-D results from the assembly of four trimeric arms, each of which contains an N-terminal collagenous domain that clusters three mannose-type Ca^2+^-dependent lectin domains (or carbohydrate recognition domains, CRDs) at the C-terminus of each arm. These trimeric CRDs facilitate multivalent, high-affinity interactions with the spike proteins on IAV, in particular the hemagglutinin (HA), a trimeric glycoprotein that mediates attachment of IAV to host cells via sialic acid (SA) receptor-mediated attachment (8, 9). This type of inhibition is highly conserved and described for SP-Ds from different animal species and is referred to as “β-type inhibition” since it involves Ca^2+^-dependent lectin-mediated binding between the CRD and high-mannose glycans present on HA. The importance of this interaction is demonstrated by loss of antiviral activity in the absence of calcium ions and by experiments with poorly glycosylated IAVs that are (more) resistant to SP-D-mediated neutralization (10–13). Upon binding to SP-D, several mechanisms of IAV neutralization are initiated that include viral aggregation, blocking IAV attachment to the epithelium, and opsonization of IAV that results in enhanced clearance of IAV by alveolar macrophages and neutrophils (7). These antiviral properties depend, to a large extent, on the state of assembly of SP-D since many studies have demonstrated that trimeric SP-D is much less active in neutralizing IAV as compared to fully assembled, dodecameric or higher order multimers of SP-D (14). Importantly, human SP-D (hSP-D) lacks the ability to bind *pandemic* IAVs due to limited glycosylation of the HA head regions. Studies in mice infected with SP-D resistant pandemic IAVs demonstrated that this leads to increased morbidity and mortality (15).

**Figure 1:**
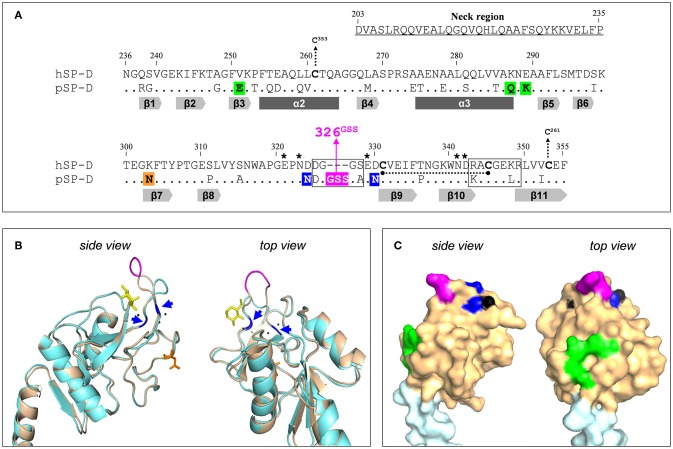
Design of the NCRD of iSP-D. **(A)** Amino acid sequence alignment of the CRD sequences from human SP-D (hSP-D, Genbank accession code: X65018) and porcine SP-D (pSP-D, AF132496). Residue numbering according to hSP-D sequence. Dashes indicate spaces that are inserted to maximize the identity across the alignment. The residues that comprise the neck region of hSP-D are underlined (residues #203-#235). Dots indicate identical residues between hSP-D and pSP-D. Dashes indicate spaces inserted to maximize the identity across the alignment. Mutated residues in hSP-D that generate iSP-D are shaded in magenta, blue and green. The *N*-glycosylation site at #303 in the CRD of pSP-D is indicated (orange box). The secondary structural regions are indicated as gray arrows (β-sheets) and dark gray bars (α-helices), deduced from the crystal structure of rat SP-A (18). SP-D groove regions (delineated), cysteines (bold) and cysteine bridges (black dotted lines/arrows), and key residues necessary for coordination of Ca^2+^-ions and hydroxyl groups of oligosaccharides (asterisks) are also indicated. **(B)** Tertiary protein structure of the neckCRD of SP-D. Shown is a ribbon diagram of the NCRD of hSP-D (blue, PDB accession no. 3G81) overlayed with that of pSP-D (brown, PDB accession no. 4DN8). A bound α-methyl mannoside as stick representation in yellow is visualized to illustrate the lectin binding pocket and 3 calcium ions are shown as black spheres. Modifications of hSP-D present in iSP-D are highlighted with deviating colors: the 326^GSS^-insertion (magenta), and the Asp324Asn and Asp330Asn mutations (blue; also indicated with blue arrows). Location of the N-linked glycan in the CRD of pSP-D is shown in orange sticks. **(C)** Surface representation of the neckCRD of pSP-D. Shown is the neck region (light blue) and the CRD (brown) of pSP-D (PDB accession no 4DN8) with porcine-specific residues mutated into hSP-D highlighted in different colors: the 326^GSS^-insertion (magenta), and the Man8-contact residues Glu251, Gln287, and Lys289 (green). Calcium ions are only partly visible on the surface (black).

The potency of SP-D to bind and neutralize IAVs varies between different animal species and it was demonstrated previously that porcine SP-D (pSP-D) exhibits distinct antiviral activity *in vitro* as compared to SP-Ds from other animal species including hSP-D and mouse SP-D (10, 16, 17). Furthermore, unlike hSP-D, pSP-D is able to bind and neutralize pandemic IAVs (10, 16). The mechanisms that underlie the exceptional IAV-neutralizing properties of pSP-D have been studied in detail and it was shown that this mainly involves distinct structural features located in the CRD of pSP-D. pSP-D is glycosylated with a highly sialylated *N*-linked glycan at Asn303 (Figure 1A) on its CRD that results in calcium-independent “γ-type inhibitor”-like activity, mediated by SA residues that interact with the conserved SA-receptor binding site located on the HA of all IAVs. *In vitro* studies with pSP-D isolated from bronchoalveolar lavage as well as studies with recombinant pSP-D demonstrate that this “γ-inhibitor” mechanism not only enhances the overall activity but that it also broadens the range of viral strains that can be inhibited, including strains that are not neutralized by hSP-D (10, 16, 19, 20). However, studies with *N*-deglycosylated full-length pSP-D showed that its distinct antiviral activity can only in part be attributed to the presence of the N-linked glycan in the CRD of pSP-D (10, 21). Sequence analysis of pSP-D aligned with hSP-D (Figure 1A) and crystallographic investigations with trimeric neckCRD (NCRD) recombinant fragments of pSP-D (22) revealed the presence of a unique tripeptide extension of the long loop in the CRD of SP-D, referred to as “326^GSS^” near the lectin site (Figures 1A–C, shaded in magenta). Molecular dynamics simulations of this fragment complexed with oligomannose showed that this flexible tripeptide adopts a more stable conformation upon sugar binding and that the insertion possibly interacts with more distal portions of the branched mannoside. Docking studies with tri-antennary octamannose underlined that with a terminal mannoside bound in the lectin site, several contacts between another arm of the oligomannose showed potential to interact with the 326^GSS^-loop. Since this loop is absent in hSP-D, this could explain the distinct Ca^2+^-mediated interactions between pSP-D and viral glycans as compared to hSP-D (22). Analysis of the crystal structure of the CRD of pSP-D also indicated that Asn324 and Asn330 might add to the flexibility of the 326^GSS^-insertion by altering the conserved structural calcium sites located between the long and short loops of the CRD, thereby increasing its ability to interact with non-terminal saccharides. In addition to the 326^GSS^-insertion at the lectin-site, several other porcine-specific residues on the surface of the CRD of pSP-D play a potential role in increased affinity of pSP-D for viral glycans. Furthermore, several non-lectin site residues on the surface of the CRD of pSP-D: Glu251, Gln287, Asn288 (conserved in hSP-D), and Lys289, form a bulge in the surface (Figures 1A,C, shaded green) that hypothetically may interact with several mannose residues from another arm of the octamannose (22). Taken together, the antiviral and crystallographic studies on pSP-D have shed light on the structural requirements that might explain its extraordinary potential to neutralize IAVs.

Previously it was shown that fully functional highly pure recombinant SP-Ds can be produced using CHO- or HEK293-cell expression systems (21, 23). This facilitates the potential value of SP-D-based drugs as a novel therapeutic or prophylactic approach to fight influenza in humans more effectively. Furthermore, recombinant approaches provide a window of opportunity to modify human-based innate defense mediators, like hSP-D, to generate derivatives that may target and neutralize a broader range of IAVs much more effectively as compared to their wildtype counterparts. SP-D-based antivirals are attractive multifunctional candidate drugs because, in addition to direct antiviral activity against a broad range of IAVs, SP-D promotes phagocytosis, down-modulates lung inflammation, and exhibits antibacterial and antifungal properties (24–26). To limit immunogenicity of protein-based therapeutic drugs, the application of human proteins is preferred. This study describes the development of “improved SP-D,” referred to as iSP-D, that is based upon full-length hSP-D and modified in its CRD by introducing 8 porcine-specific residues that are potentially important for the distinct activity of pSP-D as hypothesized in our previous studies and described above (22, 27). These mutations are highlighted in different colors in Figure 1 and include insertion of the 326^GSS^-loop (magenta), substitution of Asp324 and Asp330 by asparagine residues (blue), and introduction of the bulge-forming residues Glu251, Gln287, and Lys289 (green). After several rounds of site-directed mutagenesis, the hSP-D mutant iSP-D was successfully expressed in HEK293 cells. After purification by mannan affinity and gel filtration, the protein was characterized for its structural and saccharide-binding properties which were shown to be very similar to those of pSP-D and hSP-D. Several antiviral characteristics of iSP-D were assessed *in vitro* against a broad panel of IAVs isolated from different host species. These included binding to HA (hemagglutination inhibition assays, HAI), aggregation of IAV particles, and SP-D-mediated protection of MDCK cells against IAV infection. Overall, the strongest antiviral activity was expressed by pSP-D but iSP-D was shown to have significantly stronger antiviral activity as compared to hSP-D and proved to be active against hSP-D-resistant IAVs including pandemic IAVs. The potential of iSP-D as a novel antiviral intervention was further demonstrated by assessing its protective effects *in vivo*. To this end, a mouse IAV infection model was developed to demonstrate the therapeutic and prophylactic potential of iSP-D against infection by a clinical isolate of pandemic H1N1 virus.

## Materials and Methods

### Reagents

TOPO T/A cloning kit was supplied by Invitrogen (Breda, The Netherlands), and the Quikchange II site-directed mutagenesis kit by Stratagene (Bio-connect, Huissen, The Netherlands). Qiagen spin miniprep and midiprep kits, gel extraction kit, and proofstart DNA Polymerase were all provided by Qiagen Benelux BV (Venlo, The Netherlands). All restriction enzymes were supplied by New England Biolabs (van Westburg, Leusden, The Netherlands). The expression vector pUPE101-01 was provided by U-Protein Express (Utrecht, The Netherlands). The ToxinSensorTM Chromogenic LAL Endotoxin Assay Kit was purchased from Genscript (Piscataway, NJ, USA).

### Construction of Full-Length iSP-D

Full-length recombinant iSP-D was generated based upon the full-length hSP-D cDNA clone provided by Dr. E. C. Crouch [Washington University, St. Louis, USA; (8)]. This clone was used as a template for PCR cloning into pCR4-TOPO to introduce a BsmBI/BamHI (5′-end) and NotI (3′-end) restriction site to accommodate subcloning in expression vector pUPE-101.01, as previously described for construction of the expression clone for full-length pSP-D (21). The expression vector pUPE-101.01 is a modified version of the pTT3 expression vector (28). The full-length human SP-D clone in PCR4-TOPO was used as a template to introduce the 8 porcine-specific aa residue mutations in 5 subsequent rounds of site-directed mutagenesis by overlap extension PCR using the Quikchange II site-directed mutagenesis kit. After each round of site-directed mutagenesis, the obtained hSP-D PCR4-TOPO clone was verified by sequence analysis and used as a template to perform the next round of site-directed mutagenesis. Mutagenesis primer pairs used are listed below; all DNA sequences described are 5′ to 3′ with desired nt mutations underlined. Round 1, 326^GSS^ insert: 1FOR, GAG CCC AAC GAT GAT GGC GGC AGC AGC GGG TCA GAG GAC TGT GTG GAG with 1REV, CTC CAC ACA GTC CTC TGA CCC GCT GCT GCC GCC ATC ATC GTT GGG CTC. Round 2, Asp324Asn: 2FOR, CA GGG GAG CCC AAC AAT GAT GGC GGC AGC AGC GGG TCA GAG GAC with 2REV, GTC CTC TGA CCC GCT GCC GCC GCC ATC ATT GTT GGG CTC CCC TG. Round 3, Asp330Asn: 3FOR, GGC GGC AGC AGC GGG TCA GAG AAC TGT GTG GAG ATC TTC ACC with 3REV, GGT GAA GAT CTC CAC ACA GTT CTC TGA CCC GCT GCT GCC GCC. Round 4, Val251Glu: 4FOR, G ATT TTC AAG ACA GCA GGC TTT GAA AAA CCA TTT ACG GAG GCA CAG with CTG TGC CTC CGT AAA TGG TTT TTC AAA GCC TGC TGT CTT GAA AAT C. Round 5, Lys287Gln + Glu289Lys (both mutations were generated in a single round of site-directed mutagenesis): 5FOR, G CAA CAG CTG GTC GTA GCT CAG AAC AAG GCT GCT TTC CTG AGC ATG with 5REV, CAT GCT CAG GAA AGC AGC CTT GTT CTG AGC TAC GAC CAG CTG TTG C. After sequence verification, the pCR4-TOPO insert containing all 8 aa residue mutations were digested with BsmBI/NotI, gel purified, and transferred to pUPE101.01 as described (21). The full-length recombinant collectins pSP-D and hSP-D were constructed as published (21).

### Expression, Purification, and Biochemical Characterization of SP-Ds

All the recombinant SP-D's were expressed in HEK293 cells and purified by mannan affinity followed by gel filtration chromatography as published (21). SDS-PAGE analysis and Western blot analysis were performed as described in detail for the characterization of NpSP-D (19). Endotoxin levels were determined with the ToxinSensor LAL assay kit (Genscript, Piscataway, NJ) and endotoxin values of all SP-D preparations were below 50 pg/μg protein.

Structural characterization of purified SP-D was analyzed by Atomic Force Microscopy (AFM). SP-D stock solutions (range 1–10 μg/ml) were diluted 100x in PBS and applied on freshly cleaved mica and left for 5 min to allow for protein adherence. After rinsing with PBS followed by de-ionized water, the mica samples were left to dry at room temperature, immediately followed by AFM analysis using a Bruker Multimode IIIA AFM (112 Robin Hill Road, Santa Barbara, CA 93117, USA). AFM was performed in “Tapping Mode” using a cantilever of type Olympus AC240TS (nominal stiffness 2 N/m). Scan size was 1 × 1 μm^2^, scan rate was 1.5 Hz and images were recorded with 512 × 512 pixels. Data shown is raw data except for the default line-by-line background subtraction.

Saccharide binding affinity of all SP-Ds was tested by measuring the competition of SP-D binding to BSA/mannan-coated 96-well plates in the presence of various saccharides as previously described (29). The following mono- or disaccharides were tested: N-acetylmannosamine, L-fucose, D-galactose, D-glucose, myo-inositol, maltose, D-mannose, and fructose. Results were expressed as IC50 values, the concentrations of the various saccharides required for 50% inhibition of collectin binding to BSA-mannan. Experiments were carried out three times in duplicate.

### Influenza A Virus Strains

Hemagglutination inhibition studies were performed with 31 IAVs. These were selected based on their subtype and species of origin and included avian, swine and human IAVs of three subtypes: H1N1, H3N2, and H5N1. Details of these strains are listed in Table 2. Viruses were propagated in Madin-Darby Canine Kidney (MDCK) cells as described previously (30). After low speed centrifugation, the infectious virus titers of the culture supernatants of the infected MDCK cells were determined as described previously (30) and virus stocks were stored at −80°C until use. The three recombinant H5N1 viruses, A/Indonesia/5/05, A/VietNam/1194/04 and A/HongKong/156/97 were prepared as 6+2 reassortant strains by reverse genetics as described previously (31). The basic cleavage site in the HA protein was deleted by site-directed mutagenesis.

Viral aggregation was performed with the pandemic A/Aichi/68 (H3N2) strain which was obtained from the American Type Culture Collection (ATCC, Manassas, VA). After growth in the chorioallantoic fluid of 10-day-old chicken eggs, the virus was purified on a discontinuous sucrose gradient as described previously (20). The virus was dialyzed against PBS to remove sucrose, aliquoted, and stored at −80°C. After thawing, the viral stocks contained 5 × 10^8^ infectious focus–forming units/ml.

*In vivo* efficacy studies were performed with pandemic A/California/2009 (H1N1) E9-6714, a clinical isolate obtained from the University Medical Center Groningen (Groningen, The Netherlands). After initial amplification on MDCK cells, the virus was further propagated in the chorioallantoic fluid of 10-day-old chicken eggs as described above.

### Hemagglutination Inhibition Assay

Binding of SP-D to the viral HA and interference with binding of the virus to its receptor was assessed by HAI as previously published (16). Two-fold dilutions of SP-D or peanut agglutinin (negative control, Sigma Aldrich, Schnellldorf, Germany), which was included as a negative control, were made using Dulbecco's phosphate buffered saline containing PBS containing 1 mM of CaCl_2_ and 0.5 mM of MgCl_2_, PBS-CM (Gibco, Grand Island, USA). To 100 μl of the diluted SP-D, 2 hemagglutination units (HAU) of the respective viruses diluted in PBS-CM were added. After 1 h, 25 μl of 1% turkey erythrocytes were added. The hemagglutination patterns were read after 3 h of incubation at room temperature. The highest SP-D concentration tested was 1,000 ng/ml, except for the assays that compared activity between oligomers and trimers (only strain A/PuertoRico/8/34 (H1N1); highest concentration SP-D tested: 2,000 ng/ml). As a negative control, the experiment was also performed in PBS without CaCl_2_ to demonstrate the Ca^2+^-dependency of the SP-D activity. Statistically significant differences were calculated by two-tailed paired Student's *t*-test.

### Viral Aggregation

SP-D-mediated aggregation of IAV particles was determined with A/Aichi/68 (H3N2) and carried out as described previously (32). For both hSP-D and iSP-D, preparations of 4.0 μg/ml were mixed with a suspension of virus in a final volume of 1 ml. During stirring, the light transmission was monitored for 12 min using an SLM/Aminco 8000C spectrofluorimeter (excitation and emission wavelengths were 350 nm). Viral aggregation resulted in an increase in light transmission, and results were expressed as a percentage of control light transmission at *t* = 0. As a negative control, virus without addition of SP-D was included. Experiments were performed five times, and statistical analysis was performed with the Student's *t*-test.

### IAV Infection Reduction Assay

To investigate SP-D-mediated inhibition of infection we used an infection reduction assay as previously described (16). Briefly: 100 μl of increasing concentrations of SP-D (0–2,000 ng/ml) were incubated for 1 h at room temperature with 100 μl of the respective virus preparations containing 3,000 TCID_50_ diluted in PBS containing 1 mM of CaCl_2_ and 0.5 mM of MgCl_2_ (PBS-CM). Peanut agglutinin, which was included as a negative control, failed to reduce the number of cells infected with any of the IAVs used (data not shown). The 4 viruses used in this study were: A/Netherlands/602/09 (H1N1), A/Commonteal/Netherlands/10/00 (H1N1), A/Swine/849/93 (H3N2), and A/Mallard/NL/1/07 (H3N2). Subsequently, the mixture was transferred to MDCK cells, incubated for 1 h at 37°C and after washing, the cells were incubated for 24 h at 37°C in culture medium without trypsin to prevent secondary infection of cells by IAV. After trypsinization, the obtained cell suspensions were fixed and stained with FITC-labeled monoclonal antibodies against viral nucleoprotein (DAKOCytomation, Glostrup, Denmark). After washing, the cells were analyzed by flow-cytometry using the DIVA software.

### *In vivo* Efficacy of iSP-D Against IAV Infection

To elucidate the protective effect of iSP-D against IAV infection *in vivo*, we used a mouse infection model using 6–8 weeks old female BALBC/cOlaHsd mice (supplied from Harlan, The Netherlands). Upon arrival, the animals had an acclimatization period of one week; at the start of the experiment the mice had a weight of about 20 g. Mice were randomly allocated to experimental groups. Virus and/or SP-D (both 25 μl) was pulmonary administered with a microsprayer (PennCentury, Wyndmoore, PA, USA) just above the tracheal bifurcation after tracheal intubation with a 20G infusion cannula. Viral challenge was performed with a clinical isolate of H1N1pdm09 virus (A/California/E9/09; obtained from the Department of Medical Microbiology, Clinical Virology, University Medical Center Groningen, Groningen, The Netherlands) at a dose of 1 × 10^3^ TCID_50_ in PBS. SP-D preparations were administered at a concentration of 1 mg/ml dissolved in 5 mM Hepes (pH 7.4), 0.9% NaCl, and 2 mM CaCl_2_. Three independent *in vivo* experiments were performed with 4 groups each (group size: 6 animals) to investigate the antiviral potential of iSP-D immediately delivered after viral challenge (exp I), to compare the antiviral potential of two different batches of iSP-D with wildtype hSP-D (exp II) and to study the antiviral effect of iSP-D delivered 1 day after or before viral challenge (exp III). After initiation of the experiments, the animals were evaluated once-a-day and scored for appearance, behavior, weight and movement. An ethical end-point was considered if a cumulative weight loss of ≥15% or a weight loss of ≥10% on a single day was observed. The experiments were terminated 3 days after virus challenge and the lungs were collected for virus recovery. All animal experiments were approved by the Institutional Animal Care and Use Committee of the University of Groningen (IACUC-RUG) and were approved under number DEC 6864.

Immunohistochemical analysis was performed to investigate viral infection of the lungs using the IAV strain and route of administration as described above. Lung tissue was collected in 10% neutral-buffered formalin and fixed for 48 h followed by embedding in paraffin and sectioned at 5 μm. Sections were de-paraffinized prior to antigen retrieval with 0.1% Pronase (Merck) in PBS for 10 min at 37°C. Endogenous peroxidase activity was blocked with 3% H_2_O_2_ in PBS for 10 min and after blocking with serum (15 min, 10% Normal Goat Serum, Jackson Immunoresearch) to minimize non-specific binding, sections were incubated with either mouse anti-influenza A nucleoprotein, clone HB-65 [ATCC; (33)] or Mouse IgG2A (Thermo Fisher Scientific, Breda, The Netherlands) at 1:100 dilution for 60 min at room temperature. The incubation with DakoEnvision goat anti-mouse antibody (Agilent Technologies, Amstelveen, The Netherlands) was according to the manufacturer's protocol. Finally, slides were developed with AEC+chromogenic substrate for 30 min, counterstained with Mayer's hematoxylin for 30 s and mounted using aqueous mounting media.

Virus recovery: after weighing the lungs were homogenized in PBS (pH 7.4) and centrifuged at 1,200 rpm for 10 min. The obtained supernatants were snap-frozen in liquid nitrogen and stored at −80°C until use. Lung virus titers were determined by infecting MDCK cells grown in 96-well plates with serial dilutions of the lung homogenate supernatants. After 2 h the medium was replaced by medium containing 7.5 μg/ml TPCK-treated trypsin (Merck, Darmstadt, Germany) and plates were incubated for 72 h at 37°C, 5% CO_2_. Supernatants were then transferred to a V-bottom 96-well plate and guinea pig erythrocytes (0.3% final concentration) were added. After 2 h the virus titers in the lungs were read as the dilutions at which hemagglutination still occurred. Next, the log10 virus titer was calculated per gram of lung tissue. Statistical comparisons between experimental groups were made by two-tailed Student's *t*-test.

## Results

### Expression and Characterization of SP-D Preparations

**Figure 2:**
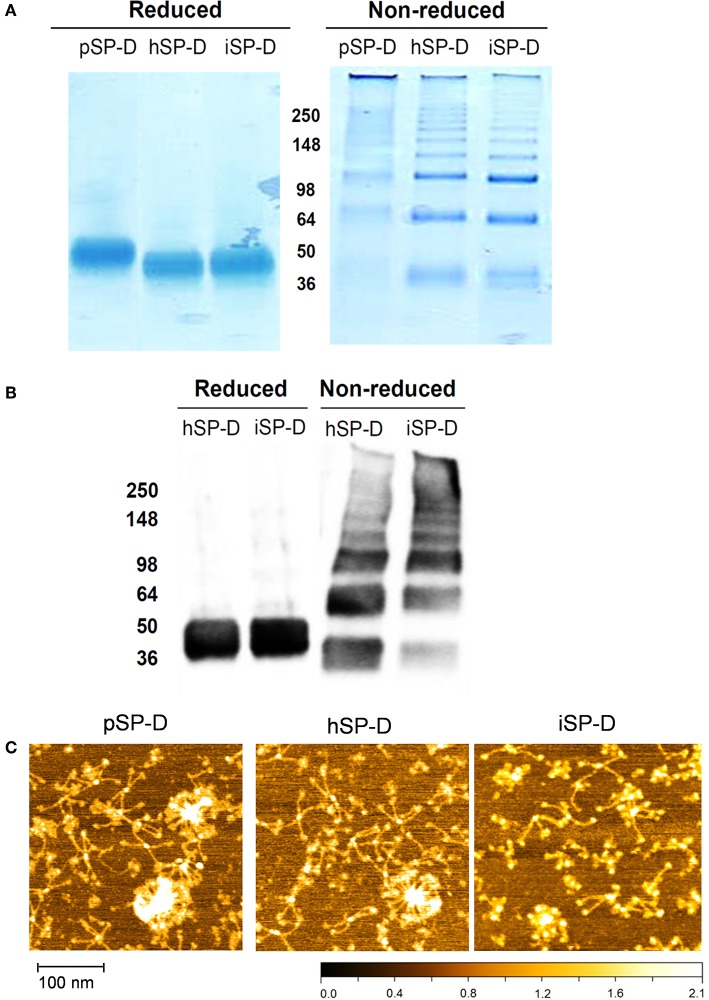
Characterization of iSP-D and comparison with other SP-Ds. **(A)** Reducing (left) and non-reducing (right) SDS-PAGE (12%) and Coomassie staining of purified full-length SP-Ds. **(B)** Western blot analysis of reduced and non-reduced hSP-D and iSP-D, followed by immunodetection with rabbit anti-human SP-D antibodies and goat anti-rabbit HRPO conjugate. The different bands visible in the non-reducing lanes are due to the presence of various oligomeric forms of SP-D that typically occurs upon non-reducing SDS-PAGE analysis of fully assembled SP-D. **(C)** Analysis of SP-Ds by Atomic Force Microscopy in ambient conditions after deposition on mica and scanning in tapping mode. The high points are colored lighter and lower points darker—full scale, black to white is 2 nm.

All full-length SP-Ds were produced using transient transfection of HEK293E cells as described previously for recombinant full-length pSP-D and hSP-D (21). The SP-D-containing cell medium was subjected to Ca^2+^-dependent mannan affinity chromatography followed by gel filtration chromatography to separate different states of SP-D oligomerization (oligomers vs. trimers). Mannan affinity chromatography did not reveal differences in Ca^2+^-dependent mannan-binding efficiency between the three recombinant SP-Ds. Transient expression of the newly developed iSP-D construct, however, resulted in significantly higher expression levels (average yield 15–20 mg/l) as compared to pSP-D and hSP-D (2.5–5 mg/l) with a similar ratio of oligomeric vs. trimeric SP-D (approximately 2:1, w/w). Analysis of the oligomeric fractions of all three SP-Ds by reducing SDS-PAGE showed a major monomeric band of ~ 50 kDa for pSP-D, while hSP-D and iSP-D migrated as slightly smaller monomers of ~ 45 kDa (Figure 2A, left lanes). Under non-reducing conditions, all SP-Ds showed multiple bands of increasing molecular weights with a distinct major band at >500 kDa, especially for pSP-D for which all bands ran at slightly higher molecular weight as compared to those from hSP-D and iSP-D (Figure 2A, right lanes). Western blot analysis of reduced and non-reduced hSP-D and iSP-D followed by immunostaining with rabbit anti-human SP-D showed identical patterns of immunoreactivity with band sizes corresponding to those obtained after Coomassie staining (Figure 2B). The oligomeric fractions of the three SP-D preparations were also subjected to structural analysis by Atomic Force Microscopy (Figure 2C). Several different oligomeric forms could be distinguished for all three SP-Ds that include dodecamers (four-armed structures), higher-order oligomers (“fuzzy-ball”-like structures) and intermediate forms. Of note, the structures present in the iSP-D preparations showed a tendency to become slightly more contracted upon AFM analysis as compared to pSP-D and hSP-D.

The saccharide binding properties of the three SP-Ds were compared using a variety of mono- and disaccharides as competitors for SP-D binding to BSA-mannan coated plates (Table 1). The IC_50_ values for each competitor were determined as concentrations (mM) required to obtain 50% reduction in SP-D-binding to BSA-mannan. The IC_50_ values were not strikingly different for all SP-Ds measured. The order of preferential glycan binding for pSP-D was almost identical to what was published previously for natural pSP-D (10). Overall, no significant differences were observed in carbohydrate binding profiles between hSP-D and iSP-D and both were comparable to what was previously determined for hSP-D (10). The only exception was the IC_50_-value for myo-inositol which was three times higher for iSP-D as compared to hSP-D, in strong contrast with pSP-D which showed highest affinity for this glycan.

**Table 1:** Comparison of saccharide selectivity of recombinant pSP-D, hSP-D, and iSP-D.

**Sugar inhibitor**	**pSP-D**	**hSP-D**	**iSP-D**
Myo-Inositol	0.9 (0.4)	5.6 (0.9)	17.3 (2.1)
Maltose	2.0 (0.2)	3.3 (0.5)	4.1 (0.1)
L-Fucose	2.2 (0.8)	3.8 (0.4)	4.3 (0.6)
Fructose	2.8 (1.1)	3.1 (0.1)	4.8 (1.3)
D-Glucose	2.8 (0.8)	4.6 (1.5)	7.8 (1.4)
D-Mannose	4.6 (1.3)	3.6 (0.5)	5.3 (2.1)
D-Galactose	6.5 (0.8)	13.5 (4.8)	13.6 (3.4)
ManNAc	8.0 (0.1)	2.2 (0.2)	3.1 (0.7)

*Microtiter wells were coated with BSA-mannan and were incubated with SP-Ds in the presence of increasing concentrations of sugars listed. Binding of collectin to BSA-mannan was measured by immunodetection as described in the Materials and Methods section. Results are expressed as concentrations (mM) of saccharide required for 50% inhibition of SP-D binding (IC_50_) to BSA-mannan. Data are averages of three experiments; SD values in parentheses*.

### Hemagglutination Inhibition of IAVs by SP-Ds

The inhibitory activity of pSP-D and hSP-D was compared to that of iSP-D by assessing the minimal concentration of SP-D required to prevent hemagglutination induced by IAV for 31 different strains (Table 2). The strains were listed according to subtype (H1N1, H3N2, and H5N1) and host species from which the strain was isolated (swine, human or avian). Overall, pSP-D was the most active inhibitor of hemagglutination by all IAVs tested with highest activity for human isolates (either H1N1 or H3N2). On average, H3N2 viruses were more susceptible to pSP-D than H1N1 viruses. In contrast to pSP-D, most of the IAVs could not be inhibited by hSP-D even at the highest dose tested (1,000 ng/ml), except for the human isolates (both H1N1 and H3N2) which were inhibited by hSP-D in the same concentration range as by pSP-D with the exception of H1N1 strain A/PuertoRico/8/34 (resistant to hSP-D). Strikingly, the hSP-D-derivative iSP-D exhibited substantial HAI activity against all IAVs with statistically significant stronger activity compared to hSP-D against 15 of the 31 H1N1/H3N2 strains tested. The overall profile of HAI values obtained for iSP-D was remarkably similar to that of pSP-D. Three different H5N1 viruses (each virus *n* = 1 only) were most resistant for inhibition by all SP-Ds and only showed some susceptibility for pSP-D, although relatively high concentrations were required to fully inhibit each of the 3 strains tested (250–500 ng/ml).

**Table 2:** Hemagglutination inhibition of IAV by SP-Ds.

**H1N1 influenza A viruses**	**SP-D preparation**			**H3N2 influenza A viruses**	**SP-D preparation**		
	**pSP-D**	**hSP-D**	**iSP-D**		**pSP-D**	**hSP-D**	**iSP-D**
**Swine (pandemic 2009)**				**Swine**			
A/Netherlands/602/09	167 (72)	>1,000 (0)	458 (473)	A/swine/Oedenrode/7C/96	63 (0)	>1,000 (0)	104* (36)
A/Califomia/4/09	161 (94)	>1,000 (0)	479 (452)	A/swine/Netherlands/849/93	16 (0)	>1,000 (0)	8* (0)
**Swine (classical)**				A/swine/Utrecht/4/85	16 (0)	>1,000 (0)	16* (0)
A/swine/Shope/11/56	458 (72)	>1,000 (0)	583 (382)	A/swine/Ukkel/1/84	44 (16)	>1,000 (0)	57* (9)
A/swine/Iowa/15/30	750 (354)	>1,000 (0)	>1,000 (0)	**Human**			
A/NewJersey/8/76	60 (5)	>1,000 (0)	83* (36)	A/Netherlands/348/07	2 (2)	4 (3)	2 (2)
**Swine (avian-like)**				A/Netherlands/548/05	1 (1)	4 (0)	1* (1)
A/swine/Netherlands/25/80	188 (108)	>1,000 (0)	375* (217)	A/Netherlands/312/03	1 (0)	5 (3)	1 (0)
A/swine/Netherlands/1/87	208 (72)	>1,000 (0)	229* (95)	A/Netherlands/35/93	16 (0)	833 (289)	52* (63)
**Human**				**Avian**			
A/Netherlands/384/06	9 (2)	89 (33)	17 (10)	A/mallard/Netherlands/19/05	26 (9)	>1,000 (0)	52* (18)
A/Netherlands/246/08	9 (6)	34 (25)	11 (4)	A/mallard/Netherlands/51/08	12 (7)	688 (541)	16 (8)
A/PuertoRico/8/34	313 (165)	>1,000 (0)	313* (165)	A/mallard/Sweden/57/03	11 (17)	>1,000 (0)	26* (9)
A/Netherlands/26/07	36 (36)	83 (36)	21* (16)	A/mallard/Netherlands/1/07	18 (12)	385 (532)	10 (4)
A/USSR/90/77	4 (4)	3 (1)	3 (4)				
**Avian**				**H5N1 influenza A viruses (*****n*** **=** **1)**			
A/mallard/Netherlands/15/05	146 (95)	>1,000 (0)	208* (72)	A/HongKong/156/97 (clade 0)	500	>1,000	>1,000
A/mallard/Netherlands/24/06	45 (30)	681 (553)	25 (11)	A/Vietnam/1194/0 (clade 1)	250	>1,000	>1,000
A/white fronted goose/Netherlands/1/07	104 (36)	>1,000 (0)	125* (0)	A/lndonesia/5/05 (clade 2.1)	250	>1,000	>1,000
A/common teal/Netherlands/10/00	83 (36)	>1,000 (0)	120* (113)				

Shown is the inhibitory activity of pSP-D, hSP-D, and iSP-D against a broad panel of IAVs isolated from different animal species (swine, human, avian) and different subtypes (H1N1, H3N2, and H5N1). Values are lowest concentrations (ng/ml) required to fully inhibit 20 hemagglutination units/ml of the respective viruses diluted in PBS. A value of “>1,000” indicates no inhibition at the highest SP-D concentrations tested. Assay procedure and details are described in Materials and Methods section. Measurements were performed in duplo (except for H5N1 viruses: n = 1) and shown is the mean of three independent experiments with standard deviations in parentheses. Statistical analysis by two-tailed paired Student's t-test (*P < 0.05 vs. hSP-D).

**Figure 3:**
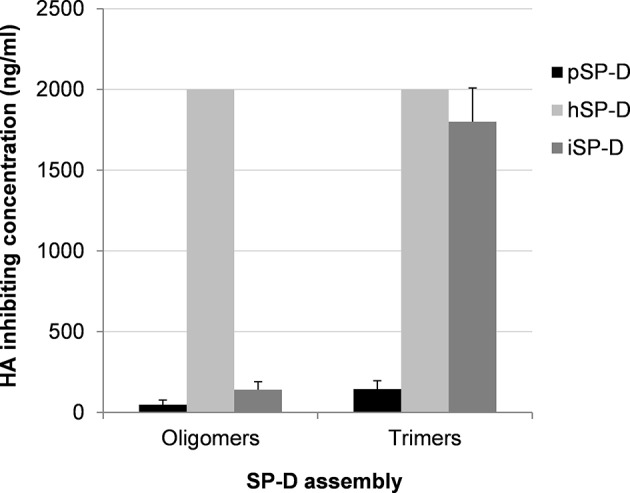
Effect of SP-D assembly on HAI activity of IAV. The inhibitory activity of iSP-D, pSP-D and hSP-D was measured for oligomeric structures and for trimeric subunit structures of SP-D. Increasing concentrations (highest concentration tested was 2,000 ng/ml) of SP-D were mixed with 2 HAU of IAV (A/PuertoRico/8/34 (H1N1) strain) and after 1 h erythrocytes were added. After 3 h incubation at room temperature, IAV-induced hemagglutination patterns revealed the lowest concentration of SP-D required to fully inhibit hemagglutination by IAV. The assay was performed in PBS containing 1 mM of CaCl_2_ and 0.5 mM of MgCl_2_. Averages are based upon five independent experiments.

Additional HAI experiments were performed using the comparatively SP-D-resistant strain A/PuertoRico/8/34 (H1N1) to determine the importance of oligomerization for SP-Ds HAI properties (Figure 3). The assay was performed with higher SP-D starting concentrations (2,000 ng/ml) as compared to the HAI data shown in Table 1. Again, fully assembled pSP-D was most profound in inhibiting this IAV strain, slightly more effective compared to iSP-D while hSP-D was not able to inhibit A/PuertoRico/8/34 (H1N1). Trimeric SP-Ds were less effective in HAI as compared to oligomeric SP-Ds. Although pSP-D trimers still retained most of their HAI activity (3-fold reduction in activity; 144 ± 52 ng/ml for trimeric vs. 48 ± 29 ng/ml for oligomeric pSP-D), the iSP-D trimers were much less effective (almost 13-fold reduction; 1,800 ± 210 ng/ml for trimeric vs. 140 ± 50 ng/ml for oligomeric iSP-D). In the case of hSP-D, trimers showed no HAI activity against A/PuertoRico/8/34 (H1N1), similar to what was found for hSP-D oligomers.

### Anti-IAV Activity of SP-Ds Assessed by Functional Assays

**Figure 4:**
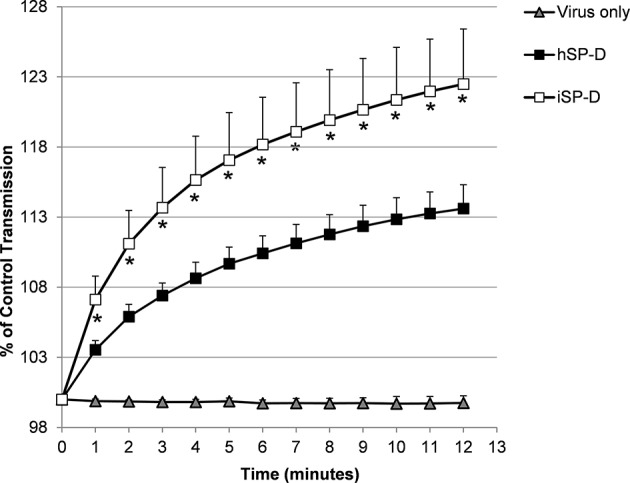
Aggregation of IAV particles by hSP-D and iSP-D. IAV particles [strain A/Aichi/68 (H3N2)] were suspended in buffer, mixed at *t* = 0 with 4.0 μg/ml iSP-D or hSP-D (control: virus only). The continuously stirred mixture was monitored for light transmission during 12 min and results are expressed as % of light transmission at *t* = 0. Measurements were performed five times and values are mean ± SEM. Statistical analysis was carried out by the two-tailed paired Student's *t*-test. *p < 0.05 (iSP-D vs. hSP-D).

The ability of SP-D to aggregate IAV particles was determined using the pandemic A/Aichi/68 (H3N2) strain. Virus incubated in the presence of 4.0 μg/ml hSP-D resulted in aggregate formation as demonstrated by a gradual increase in light transmission (black squares, Figure 4). In the presence of the same amount of iSP-D, aggregation was significantly increased (75% increase after 12 min as compared to hSP-D (white squares, Figure 4). As a control, incubation of virus in buffer only did not result in any aggregation.

**Figure 5:**
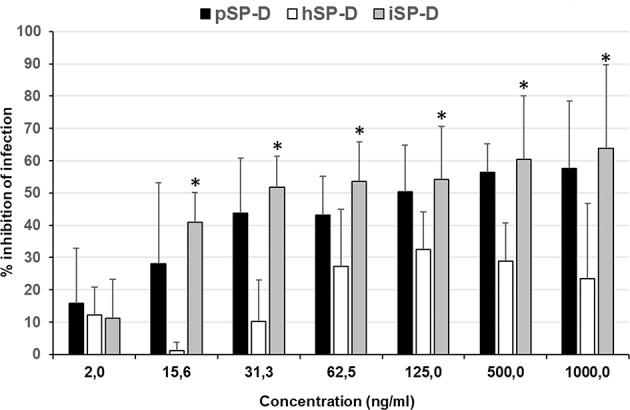
Neutralization of IAV infection by SP-Ds. The infection reduction assay was used to measure and compare the ability of hSP-D, iSP-D, and pSP-D to inhibit IAV infection of MDCK cells. Increasing concentrations of SP-D were mixed with 3,000 TCID_50_ of 4 different IAV preparations: 2 H1N1 subtype IAVs and 2 H3N2 subtype IAVs. After 1 h pre-incubation at room temperature, the mixtures were added to MDCK cells, incubated for 1 h at 37°C, and after washing, the MDCK cells were incubated for 24 h at 37°C in medium without trypsin to prevent secondary rounds of infection by IAV. Cell suspensions, obtained after trypsinization, were fixed and stained for viral nucleoprotein. After washing the cells were analyzed by flow-cytometry using the DIVA software. Reduction of infectivity was expressed as the relative number of cells that became infected according to the formula: % reduction = 1–(% infected cells in presence of SP-D/% infected cells without SP-D)*100%. Shown is the average value of the 4 different strains analyzed. Statistical analysis was carried out by the two-tailed paired Student's *t*-test. *p < 0.05 (iSP-D vs. hSP-D).

An infection reduction assay was used to determine the capacity of SP-D to protect MDCK cells from infection by IAV with 4 different strains (2 strains of H1N1 subtype and 2 strains of H3N2 subtype); the anti-IAV activity was expressed as % inhibition of infection as compared to IAV infection in absence of SP-D (Figure 5). Peanut agglutinin, which was included as a negative control for SP-D, did not reduce the number of cells infected with any of the IAV strains used (data not shown). In contrast, pSP-D inhibited infection of MDCK cells by all strains tested and the average minimal dose of pSP-D required to obtain at least 50% inhibition of infection by IAV was approximately 30 ng/100 μl. In general, higher doses of pSP-D (up to 1,000 ng/μl) resulted in slightly higher percentages of inhibition and in this cellular infectivity assay, none of the viruses tested were fully inhibited by pSP-D. Compared to pSP-D, the activity of hSP-D was substantially lower at all concentrations tested and the average highest percentage of inhibition observed was approximately 30%, even at the highest dose of hSP-D. Strikingly, the infection reducing activity of iSP-D was almost similar to that of pSP-D and substantially higher compared to that of hSP-D. Compared to hSP-D, the activity of iSP-D was statistically significant higher for all concentrations measured except the lowest concentration tested (2.0 ng/ml).

### *In vivo* Validation of the Antiviral Activity of iSP-D

**Figure 6:**
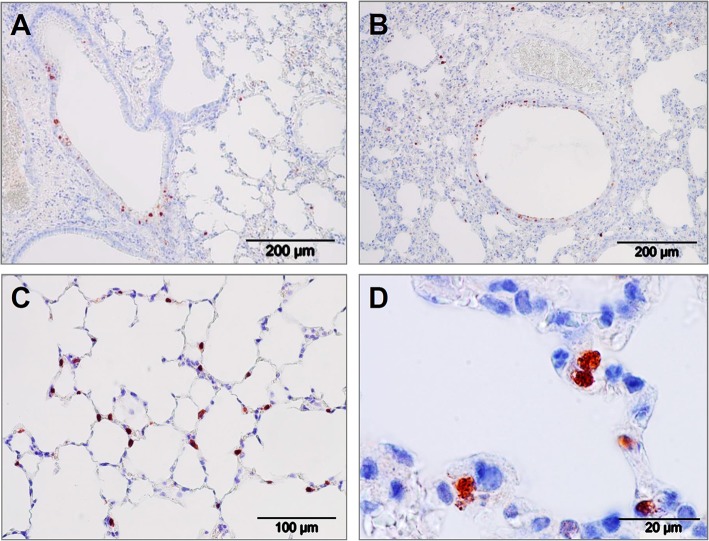
Lung immunohistology of pandemic IAV infection mouse model. Pattern of pandemic IAV-infection in the lungs from mice used as a model for antiviral efficacy studies with iSP-D. Photomicrographs of hematoxylin-stained tissue sections and immunohistochemically stained sections for detection of influenza viral nucleoprotein from mice infected with a clinical isolate of pandemic A/California/E9/09 (H1N1) at a dosis of 1 × 10^3^ TCID_50_ in PBS. After intubation, the virus was administered by microspraying just above the tracheal bifurcation. Three days post-infection, lungs were collected, fixed, embedded in paraffin and further processed as described in the Materials and Methods section. Viral antigen is stained reddish brown against a hematoxylin-stained blue background and shown for different areas of the lower respiratory tract: **(A,B)** bronchiolar epithelial cells, **(C,D)** alveolar epithelial cells. Scale bars as indicated in each panel.

The virus inhibiting potential of iSP-D *in vivo* was assessed by using a mouse model for influenza and to demonstrate the protective potential against a human-derived virus strain, a clinical isolate of pandemic H1N1 virus (E9-6714, named H1N1pdm09 in the following) was used for inoculation. The optimal dose and route of administration (either intranasal, intratracheal, or intratracheal using a microsprayer) for this pandemic strain was first determined in a pilot experiment (4 animals/group). Results indicated that a dose of 10^3^ TCID_50_ delivered by the use of pulmonary microsprayer inoculation resulted in rapid weight loss and broader distribution of the virus over the lungs as determined by immunohistochemical analysis of lungs 3 days after infection with IAV; representative images are shown in Figure 6. Viral infection was observed in bronchiolar epithelial cells (Figures 6A,B) as well as in in alveolar epithelial cells (Figures 6C,D). It should be noted that the immunohistochemical analysis could not be used in a quantitative fashion since measured differences in lung virus titers and weight development were not noticeable by immunostaining of the virus *in situ*.

**Figure 7:**
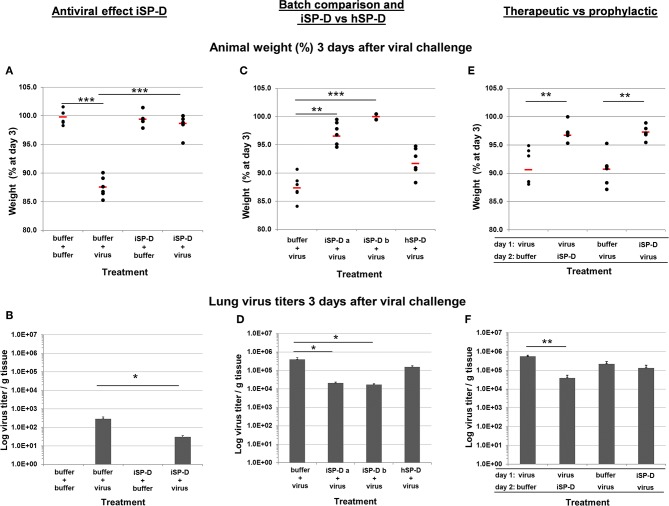
Validation of iSP-D as antiviral agent against pandemic IAV infection in mice. The protective potential of iSP-D against pandemic IAV infection *in vivo* was assessed using the model as described in the materials and methods section and in Figure 6. Three different experiments were executed with 4 different conditions within each experiment; group size was *n* = 6 per condition. Administration of iSP-D or hSP-D and viral challenge (A/California/E9/09 (H1N1) strain, 1 × 10^3^ TCID_50_) as described in the Materials and Methods. Three days after infection the average animal weights (% of weight at day 0) and average virus titers (log virus/g lung tissue) were determined for all experimental groups. Experiment I, **(A)** (animal weight) and **(B)** (virus titers): antiviral effect of iSP-D-delivery (25 μg/animal) immediately followed by viral challenge. Experiment II, **(C)** (animal weight) and **(D)** (virus titers): comparison of antiviral activity between 2 different batches of iSP-D and between iSP-D and hSP-D (5.0 μg/animal). Experiment III, **(E)** (animal weight) and **(F)** (virus titers): the antiviral effect of iSP-D (25 μg/animal) delivered 1 day after or 1 day before viral challenge. Statistical comparisons between experimental groups were made by Student's two-tailed paired *t*-test. *p < 0.05; **p < 0.005; ***p < 0.0005.

Using the parameters described above, this infection model was used to determine the protective value of iSP-D *in vivo* (Figure 7). The first experiment was executed by administering 25 μg of iSP-D, immediately before inoculation with IAV, and included 3 control groups that received buffer only, virus only or iSP-D only (6 animals/group). All animals were monitored for changes in body weight and results showed an average weight loss of 12.5% over 3 days in the mice that were treated with buffer followed by inoculation with virus (Figure 7A), just below the ethical end point which was set at a cumulative weight loss of >15%. In contrast, the mice that were first treated with iSP-D, followed by inoculation with the same 10^3^ TCID_50_ dose of H1N1pdm09, did not show any weight loss. Administration of iSP-D only did not result in any changes in body weight as compared to treatment with buffer only. In line with protection from infection-induced weight loss, the iSP-D-treated mice were also partially protected from virus replication in the lungs (Figure 7B) since the average lung virus titer in iSP-D-treated mice was approximately 10 times lower compared to non-treated mice.

Next, we evaluated whether different batches of iSP-D were equally effective in protecting from influenza disease symptoms and virus growth in the lungs. In addition, we compared the activity of iSP-D with that of hSP-D (Figures 7C,D, respectively). In this experiment a lower dose of SP-D was administered (5.0 μg/animal) to monitor differences in protective activity between iSP-D and hSP-D more adequately. Mice that were infected with IAV after treatment with buffer had a mean weight loss of 12.6% by day 3. In contrast, the mice treated with iSP-D batch-a had a maximal weight loss of 5.4% and 3 out of 6 animals did not lose weight at all (*p* < 0.0005 vs. control group). The iSP-D batch-b was slightly more effective since none of the mice developed more than 0.5% weight loss (*p* < 0.00005 vs. control group). The group that received treatment with hSP-D, prior to infection, resulted in no statistically significant difference in weight loss compared to the buffer-treated control group (Figure 7C). The pattern of observed animal weight loss resulting from treatment with or without iSP-D or hSP-D was also reflected by lung virus titers (Figure 7D). Whereas, lung virus titers were significantly reduced upon treatment with iSP-D (batch-a or -b) as compared to the control group (buffer only), no statistically significant reduction was observed when hSP-D was administered prior to infection.

In the third *in vivo* validation experiment we aimed to test the prophylactic and therapeutic potential of iSP-D by expanding the time-interval (24 h) between drug administration followed by infection (prophylactic use) and by reversing the order of administration (infection followed by iSP-D treatment) to test whether iSP-D contributes to recovery from previously established IAV infections (therapeutic use). As illustrated in Figure 7E, 3 days after virus challenge, treatment with iSP-D either 24 h before infection or 24 h after infection, resulted in significantly reduced weight loss as compared to control treatment (virus/buffer or buffer/virus). Determination of lung virus titers showed that the protective effect of iSP-D was most profound upon therapeutic application, resulting in a significantly reduced virus titer (14 times lower) as compared to the buffer control group (Figure 7F). Only a slight statistically nonsignificant reduction in virus titer was observed when iSP-D was administered 24 h before virus challenge as compared to buffer.

## Discussion

The innate immune response is considered crucial for containment of viral spread at early stages of infection and many previous studies, *in vitro* and *in vivo*, have shown that SP-D is known to play an important role in protection against IAV infection (4). Animal species-specific differences in anti-IAV activity of SP-D have been demonstrated and may result from variations in key amino acid residues located in the CRD near the lectin binding site which facilitates interactions with glycans exposed on the surface of IAV (11, 34). Porcine SP-D, in particular, has unique structural features that sets it apart from all other SP-D species. An N-linked sialoglycan in the CRD of pSP-D facilitates interactions with the sialic acid receptor of HA, the spike protein of IAV responsible for viral attachment to the host epithelium. Follow-up structural studies with deglycosylated NCRD fragments of pSP-D revealed that its CRD contains a unique tripeptide loop (326^GSS^), that most likely contributes to the distinct lectin-mediated activity of pSP-D (22). Crystallography and docking studies with octamannose also revealed 5 other residues that are hypothetically involved in generating profound anti-IAV activity as displayed by pSP-D: Asn324 and Asn330 might increase flexibility of the 326^GSS^ loop, while Glu251, Gln287, and Lys289 are unconserved residues that could make contact with distal parts of lectin-bound octamannose (unpublished observations). To investigate to what extent these porcine-specific residues contribute to the distinct lectin-mediated antiviral activity of pSP-D, we generated iSP-D, a hSP-D-based mutant that has 8 amino acid residues in its CRD modified according to the pSP-D sequence (Figure 1).

After several rounds of site-directed mutagenesis on the full-length wildtype hSP-D clone, iSP-D was successfully expressed in HEK293 cells and purified from the cell-free medium as described for other recombinant SP-Ds (21, 22). The molecular structure of iSP-D, as analyzed by SDS-PAGE and AFM, was very similar to that of hSP-D and only small differences were observed with regard to the spatial arrangement of the oligomeric arms of iSP-D (slightly more condensed compared to that of pSP-D and hSP-D). Since the collagen domain of iSP-D is identical to that of hSP-D, this is most likely due to subtle differences in sample preparation for AFM analysis. iSP-D was also characterized by investigating its saccharide binding properties for various mono- and disaccharides (Table 1). The overall binding affinity profile of iSP-D was very similar to that of hSP-D, the only difference observed was its decreased affinity for myo-inositol. Interestingly, pSP-D showed highest affinity for this monosaccharide but substitution of porcine-specific residues into hSP-D did not generate preferential recognition of this ligand by iSP-D. Previously, it was shown that mutation of residues that flank the primary carbohydrate recognition site can have an impact on the saccharide binding selectivity (35–37). In the case of iSP-D, crystallographic analysis of the NCRD of iSPD in combination with modeling and/or docking studies will be required to determine the mechanism that underlies the difference in affinity observed for myo-inositol. It most likely involves the additional loop 326^GSS^ and/or the substituted residues Asn324 and Asn330. Of note, glycan array studies with a library of natural and synthetic oligosaccharides would be required to provide more insight on the lectin binding selectivity and possibly improved affinity of iSP-D toward larger highly branched glycans, as present on the HA of IAV.

Screening the HAI activity of iSP-D against a panel of IAVs with different subtypes and host origins enabled us to determine whether the modifications that were engineered into hSP-D enhanced its antiviral potential. Furthermore, screening helped to determine if the mutant iSP-D displayed distinct inhibitory properties against IAV to the level of what was demonstrated previously for pSP-D (10, 16). As shown in Table 2, the different IAV strains showed varying degrees of susceptibility to SP-D-mediated neutralization for all three SP-Ds tested. However, it was demonstrated for all strains that the mutant iSP-D exhibited an overall strong HAI activity, except for the H5N1 strains which among all IAVs tested were most resistant against all SP-Ds, including pSP-D. Strikingly, the antiviral activity profile of iSP-D was highly similar to that of pSP-D and for more than half of all the 31 strains tested, there was a statistically significant increase in HAI activity of iSP-D compared to hSP-D. Clearly, these findings confirmed our hypothesis that the selected 8 porcine-specific residues were important in contributing to the distinct anti-IAV activity of pSP-D since genetic modification of hSP-D with these 8 residues, strongly improved the moderate antiviral activity of wildtype hSP-D. Moreover, the “porcinized” hSP-D mutant iSP-D expressed HAI activity to a degree that is in the range of that of pSP-D. Interestingly, the improved antiviral properties of iSP-D were most pronounced against those IAV strains that were designated as resistant against inhibition by hSP-D (no HAI observed with highest concentration tested) and these included pandemic and avian(-like) strains known to be resistant for inhibition by hSP-D (15, 38). Most likely, the improved activity of iSP-D against these strains resulted from enhanced binding affinity to the small number of mannosylated glycans present on these strains. In contrast, the more recent human seasonal H1N1 and H3N2 isolates were most susceptible for HA inhibition by all SP-Ds. These observations are in line with previous studies that showed that in time strains circulating in the human population acquire more glycans on the head region of the viral HA, most likely due to shielding the HA from antibody-mediated neutralization (12). As a consequence, these IAVs are more prone to lectin-mediated binding and subsequent enhanced neutralization, in contrast to isolates obtained shortly after introduction in the human population which possess fewer glycans and are thus more resistant against neutralization by hSP-D.

Many studies have demonstrated the importance of the degree of oligomerization for the efficacy of SP-D to bind and neutralize bacteria and viruses, including IAV (39, 40). In general, multivalent interactions by (multi)dodecameric SP-D with multiple IAV particles result in more efficient aggregation and neutralization of IAV as compared to SP-D trimers, also demonstrated for pSP-D and hSP-D against a broad panel of IAVs (16). To investigate possible differences in HAI activity between trimeric pSP-D, hSP-D and iSP-D, the relatively SP-D-resistant A/PuertoRico/8/34 (H1N1) strain was used while oligomeric SP-Ds were included as control. As expected, the oligomeric SP-Ds were most effective and activity was in the order pSP-D > iSP-D > hSP-D (Figure 3). In accordance with previous studies, this IAV strain was found to be resistant for inhibition by oligomeric hSP-D and, not surprisingly, the generally less active trimeric form of hSP-D also showed no activity with the highest doses of hSP-D tested (2,000 ng/ml). Interestingly, the oligomeric forms of pSP-D and iSP-D showed activity in the same range (50–150 ng/ml) against A/PuertoRico/8/34 (H1N1), but there was a major difference in HAI activity between their trimeric forms: trimeric iSP-D was 13-fold less active than trimeric pSP-D. This can, at least in part, be explained by the presence of the N-glycan in the CRD of pSP-D which is absent in hSP-D. The glycan's presence would allow for both β- and γ-inhibition of the virus, which may be critical for activity of the trimeric forms, whereas the γ-inhibition may play a less critical role in determining the activity of oligomeric forms. However, even iSP-D trimers showed activity against this hSP-D-resistant strain and this underlines the potential of trimeric iSP-D, or even collagen-free NCRD fragments, as an hSP-D-based antiviral with profound broad-range anti-IAV activity. To verify this, additional studies with trimeric forms of iSP-D against a larger collection of IAVs will be required. The viral aggregation properties of (oligomeric) iSP-D were also examined and compared to those of hSP-D (Figure 4). Full-length SP-D is able to bind and aggregate virus particles, a well-known and important protection mechanism that helps to prevent attachment of infectious IAV particles to the lung epithelium and that promotes clearance by phagocytosis (41). By the use of the pandemic strain A/Aichi/68 (H3N2) it was determined that iSP-D exhibits significantly stronger IAV aggregation properties as compared to hSP-D. Most likely this is due to the distinct lectin-mediated affinity of iSP-D for the N-linked oligosaccharides present on the surface of IAV (HA glycosylation), resulting in faster aggregation and/or formation of larger viral aggregates. Unfortunately, we were not able to include pSP-D as reference although it was previously established that pSP-D, like iSP-D, is more effective in viral aggregation as compared to hSP-D (22).

In addition to HAI and viral aggregation, we also examined the potential of iSP-D to reduce IAV infection with a previously established IAV infection reduction *in vitro* assay using MDCK cells (16). With this infection model, we tested and compared the antiviral activity of pSP-D, hSP-D and iSP-D measured against 4 IAV strains. It should be noted that for unknown reasons the dose-dependency using this assay was restricted, regardless of the IAV strain or SP-D preparation used, and none of the viruses could be fully inhibited with the highest doses of SP-D tested. Despite these assay limitations, it was clearly established that compared to hSP-D, iSP-D was substantially more effective and reached maximum inhibition levels much higher than hSP-D. The inhibition profile is comparable to that of pSP-D. Based upon these observations it can be concluded that compared to hSP-D, iSP-D exhibits superior IAV neutralizing activity *in vitro*, not only demonstrated by HAI and viral aggregation, but also by its strongly improved ability to protect cell monolayers from infection by IAV.

Overall, pSP-D appears slightly more active against all IAVs tested as compared to iSP-D and this could be due to the presence of the N-linked glycan in the CRD of pSP-D (lacking in iSP-D) that was previously shown to contribute to the IAV neutralizing properties of pSP-D (10, 19, 27). Consequently, the antiviral properties of iSP-D might be further improved by N-glycosylation of its CRD with a complex sialic acid-rich oligosaccharide. It should be noted, however, that previous studies have demonstrated that such modifications in the CRD of hSP-D would not necessarily result in improved inhibitory properties against IAV and likely involves additional, yet unknown structural prerequisites present on the CRD of pSP-D (21). Since iSP-D, in contrast to hSP-D, contains several porcine-specific residues that have an impact on its interaction with IAV, it can be speculated that iSP-D might be more suitable as a template to perform N-glycosylation modification studies. Ultimately this could result in the design and production of a highly active hSP-D-based glycosylated mutant that exhibits both β-inhibitor and γ-inhibitor-like properties, as demonstrated for pSP-D.

The importance of SP-D in protecting against IAV infection *in vivo* has been demonstrated by the use of SP-D knockout mice (SP-D -/-) that, upon infection by IAV, show increased lung virus titers and weight loss as compared to wildtype mice (42, 43); pulmonary instillation or over-expression of SP-D can correct these effects (44, 45). The efficacy of SP-D-mediated clearance, however, depends on the level of glycosylation of the viral spike proteins (12, 46). Pandemic IAV strains, in particular, are associated with low expression of N-glycans in the head region of their HAs and consequently, these strains are fairly resistant for lectin-mediated binding and subsequent neutralization by SP-D (38). Therefore, the efficacy of iSP-D *in vivo* was tested with the 2009 pandemic H1N1 virus, not only because pandemic IAVs are of special interest with regard to their implications for human health, but also because this virus is resistant to neutralization by endogenous wildtype SP-D. Weight loss of infected mice was rapid and consistent and is considered to be an important and sensitive indicator of diseases symptoms in mice. Immunohistochemical analysis was performed on IAV-infected mice and sections were stained for the presence of viral antigens. Results indicated that H1N1pdm09 infection in mice induced bronchiolitis but also alveolitis, with virus-staining in epithelial cells lining the bronchioles and the alveoli (Figure 6). In a study by Qi et al. the same was observed in a mouse infection model and it was speculated that pandemic IAV-induced lower respiratory pathology is associated with demonstrated low binding activity of SP-D for pandemic IAVs (15). Moreover, these observations also mimic closely pulmonary infections in humans caused by pandemic IAV (e.g. 1918, 1957, 1968, and 2009) characterized by distinct, substantial pathological changes of the lower respiratory tract, associated with widespread infection of bronchiolar as well as alveolar epithelial cells (47, 48).

With this pandemic IAV mouse infection model, the protective potential of iSP-D was investigated in three different experiments. The first experiment showed that H1N1pdm09 infection in mice induced profound weight loss and substantial lung virus titers 3 days after challenge. If infection was immediately preceded by treatment with iSP-D, animal weight loss was completely prevented and lung virus titers were significantly reduced, the first *in vivo* evidence for the protective value of iSP-D against pandemic IAV. Administration of iSP-D alone did not result in any changes in weight and behavior, a preliminary indication that iSP-D does not have a direct toxic effect after pulmonary delivery in mice. The second experiment not only confirmed the protective effects, but also showed that iSP-D batch-to-batch variations are very small. Furthermore, by including a condition in which hSP-D was administered instead of iSP-D, it was demonstrated that hSP-D could not prevent infection-induced weight loss while only a small reduction in lung virus titers was observed, consistent with previous observations *in vitro* that showed resistance of A/California/E9/09 (H1N1) for neutralization by hSP-D. In contrast, iSP-D gained antiviral activity due to the pSP-D-inspired mutations. Surprisingly, the average lung virus titers obtained 3 days after infection were considerably higher in the second (and third) experiment as compared to the first experiment. This inconsistency remains difficult to explain since the same batch, amount and volume of virus was used and the route of administration was identical in all experiments. The third experiment was designed to mimic more closely the use of iSP-D as an antiviral for prophylactic or therapeutic applications by expanding the interval between virus challenge and iSP-D treatment (24 h). When treated with buffer, either 1 day before or after virus challenge, all mice showed consistent weight loss which was on average 9% at day 3 after infection. In contrast, treatment with iSP-D 24 h before or after infection resulted in only minor weight loss (2.7–3.3%) and these data suggest that iSP-D can significantly reduce clinical symptoms caused by pandemic IAV infection in mice, either prophylactically or therapeutically. There was a major difference, however, in lung virus titers. These were significantly lower in the group that received treatment 1 day after inoculation and were only slightly reduced when iSP-D was administered 1 day before inoculation. Although the mechanism is not fully understood, this could be due to the anti-inflammatory properties of iSP-D. When administered prophylactically, iSP-D may contribute to reduction of disease symptoms caused by IAV infection but less capable in inhibiting viral replication, in contrast to therapeutic application that results in significant reduction in weight loss as well as lung virus titers. Another explanation for the observed differences can be due to degradation of iSP-D (e.g., by pulmonary proteases) during the 24 h before virus challenge. The slight reduction in lung virus titers seem to contradict the observed prevention of weight loss. A possible explanation is that this could be due to the anti-inflammatory effects exerted by iSP-D and subsequent reduction of disease symptoms. In a follow-up study we aim to include immunohistochemical analysis of pulmonary inflammatory responses in order to determine how these are affected after treatment with iSP-D.

The encouraging disease-reducing effects exerted by iSP-D, especially in a therapeutic setting when only a single dose is administered after infection with pandemic IAV, underline the potency of iSP-D as a novel antiviral drug against a broad range of IAV strains. As a novel inhalation drug, iSP-D could contribute to reduction of disease severity, especially in settings of pandemic outbreaks. This could prove important to protect against potentially lethal IAV variants to bridge the time required for the production of effective vaccines against such newly emerging IAVs. The effect of iSP-D on lung virus titers in the different challenge models is relatively modest. However, the beneficial effects of iSP-D on the weight of infected animals are very clear and suggest that the anti-inflammatory properties of iSP-D contribute to the reduced morbidity. The protective effects of iSP-D can be further enhanced by dose-optimization, drug formulation, and repeated administration. Although iSP-D can be considered as a human-based protein, such investigations should run in parallel with safety studies with regard to possible (immuno)toxic effects. It should be noted that, since iSP-D only has 8 residues changed as compared to wildtype hSP-D (of a total of 358 amino acids in a single polypeptide), severe complications due to immunogenicity are less likely to occur compared to other, non-human protein-based, antivirals.

Additional modifications of the molecular properties of iSP-D can also be considered (e.g., N-glycosylations) to further enhance the virus-neutralizing properties of iSP-D. Taken together, our findings suggest that recombinant SP-D-based compounds could generate a new class of versatile antiviral drugs against seasonal and pandemic IAV and, considering their demonstrated broad-range antimicrobial properties, with potential for effective treatment of pulmonary infectious diseases caused by other respiratory pathogens in humans.

## Data Availability Statement

All datasets generated for this study are included in the article/supplementary material.

## Ethics Statement

The animal experiments performed under this protocol were approved by the Committee for Animal Experimentation (DEC) of the University Medical Center Groningen, according to the guidelines provided by the Dutch Animal Protection Act (permit number DEC 6864).

## Author Contributions

ME and HH designed the study. ME purified and characterized the SP-D preparations, and wrote the manuscript. MLBH, GR, MW, and KH designed and executed the *in vitro* virus experiments. MR assisted in designing iSP-D. MH assisted in expression of recombinant proteins. PK and MT performed the immunohistochemical experiments. MH performed the AFM experiments. TM and AH designed and executed the *in vivo* experiments. HH, GR, KH, and AH reviewed and improved the manuscript. HH supervised the study.

### Conflict of Interest

MH was employed by company U-Protein Express B.V., Life Science Incubator. The remaining authors declare that the research was conducted in the absence of any commercial or financial relationships that could be construed as a potential conflict of interest.

## Abbreviations

326^GSS^: pSP-D-specific insertion of Gly-Ser-Ser adjacent to residue Gly-326 of the SP-D consensus sequence
CRD: carbohydrate recognition domain
HA: hemagglutin
HAI: hemagglutination inhibition
HAU: hemagglutinating units
hSP-D: human SP-D
IAV: influenza A virus
iSP-D: improved SP-D
MDCK: Madin-Darby canine kidney
NCRD: neck region plus CRD from SP-D
pSP-D: porcine SP-D
SA: sialic acid
SP-A: surfactant protein A
SP-D: surfactant protein D.
